# Bird Migration Advances More Strongly in Urban Environments

**DOI:** 10.1371/journal.pone.0063482

**Published:** 2013-05-08

**Authors:** Piotr Tryjanowski, Tim H. Sparks, Stanisław Kuźniak, Paweł Czechowski, Leszek Jerzak

**Affiliations:** 1 Institute of Zoology, Poznań University of Life Sciences, Poznań, Poland; 2 Fachgebiet für Ökoklimatologie, Technische Universität München, Freising, Germany & Institute for Advanced Study, Technische Universität München, Garching, Germany; 3 Faculty of Engineering and Computing, Coventry University, Coventry, United Kingdom; 4 Institute for Tourism and Recreation, State Higher Vocational School in Sulechów, Sulechów, Poland; 5 Faculty of Biological Sciences, University of Zielona Góra, Zielona Góra, Poland; Hungarian Academy of Sciences, Hungary

## Abstract

Urbanization has a marked effect on the reproduction and other ecological and behavioural traits of many living organisms, including birds. In migrant birds, survival and reproductive output is influenced by the (mis)synchronization of arrival with the availability of resources. Many recent studies have shown that arrival timing is related to temperatures *en-route* and at destination. Because urban areas are “heat islands”, with higher temperatures that influence earlier vegetation and invertebrate development, this should favour earlier arrival of migrant birds to cities rather than to rural areas. In this paper, we analysed differences between urban and rural habitats in mean dates and trends of first arrival dates of 18 species of migratory bird species in western Poland during 1983–2010. For many individual species, and overall, mean first arrival date was significantly earlier in rural areas than in urban areas (significant for 11 species). However, the trend towards earlier first arrival dates was stronger in urban areas for 15 of the 18 species (significantly stronger in four species). Consequently, arrival dates in urban areas are fast approaching, or have now matched or passed those in rural areas. These findings suggest that recent environmental changes may have more rapidly changed the migratory habits of birds occupying urban habitats than those occupying rural habitats.

## Introduction

Urban development is increasing across the Globe and having major impacts on animal life-histories [1]–[4]. Sometimes changes in the environment are so extreme that adjustment to novel urban environments may even require genetic adaptation [2], [4]. Responses to environmental pressures include the need to maintain synchrony in specific time windows. Among birds, the timing of migration and, in consequence, time of reproduction may be critical [5], [6]. To date, the timing of when birds return to their breeding area has been a key component of studies of the impact of climate change upon bird populations, because arrivals are strongly related to temperature [7]. At the landscape scale, arrivals to warmer habitats/places should be earlier than to cooler ones. However, to the best of our knowledge, there is a lack of data suitable to investigate this theory. A good example of warmer environments are cities, characterised by higher temperatures than their surroundings and hence sometimes called “heat islands”. Moreover, it has recently been noted that global increases in temperature may be particularly strong in cities [8]–[10]. Because of higher temperature, urban environments may also supply an abundance of food due to higher primary productivity, a longer growing season, and intentional (bird feeders) and unintentional (waste food) feeding by humans [4], [11]. In contrast, cities may have reduced food availability of several important arthropod prey [12]. However, most studies and reviews have shown earlier plant phenology in urban areas [11]–[15], and consequently invertebrates also develop earlier and faster [14]–[17]. All of these environmental changes should positively influence bird arrival timings, and therefore we may hypothesise that cities will be associated with an earlier arrival of migratory birds than rural habitats. Although this idea is simple it is surprising that to date, to the best of our knowledge, this has not been investigated.

On the other hand, urbanization is designed to generally lead to an environment favourable for humans but it can simultaneously result in a host of environmental problems, including the loss of biodiversity and ecosystem services [9], [12]. There has been a discussion recently on which species gain, and which lose, from pressures brought about by urbanization [4], [5], [9], [17]–[20]. Changes in phenology are also seen as a reaction to avoid population decline, and species which have adapted to temperature have had healthier population sizes [21]. Furthermore, urban habitats cover increasingly large fractions of the Earth, with further increases predicted [3], [9]. Recently, the proportion of humans living in cities exceeded 50% for the first time.

The objective of this study was to assess whether urban or rural habitats were occupied first by returning migrant birds in spring and whether arrival patterns were changing. The study was undertaken in medium-sized Polish cities and surrounding rural habitats. However, because urban environments have a strong effect on changes in climate at the global scale [22], the processes described in this paper may have a wider importance.

## Results

### Mean First Arrival Dates

**Figure 1:**
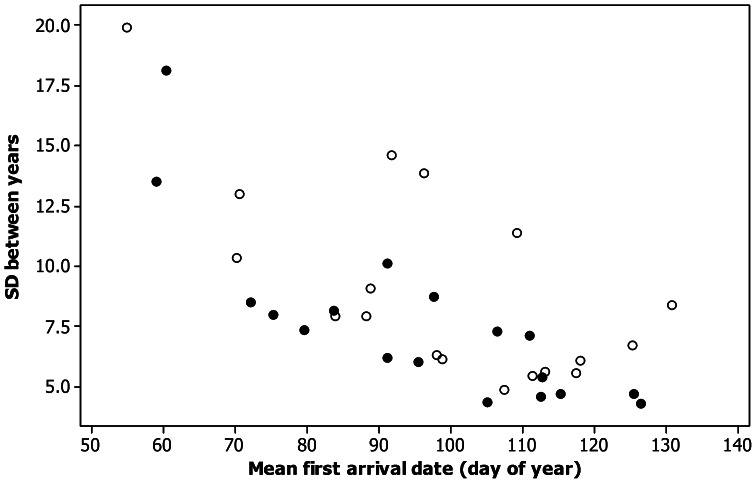
The relationship between standard deviation (SD) of first arrival dates and mean first arrival date (shown as day of the year, 1 =  Jan 1 etc.) for 18 species recorded at rural sites (solid symbols) and urban sites (open symbols) in Western Poland.

Mean first arrival dates in rural and in urban areas for common years are summarised in Table 1. As shown in previous studies, and based on all years recorded, there was a negative correlation between mean first date and standard deviation between years for both rural (r_16_ = −0.81, p<0.001) and urban records (r_16_ = −0.68, p = 0.002), i.e. early arriving species had greater between years variability and vice versa (Figure 1). A paired t-test on species standard deviations from rural and urban data in common years confirmed that urban arrivals were significantly more variable than rural arrivals (t_17_ = −2.34, p = 0.032). Based on common years, differences between mean first arrival dates in rural compared to urban areas ranged from 13 days earlier (white stork) to 5 days later (wood pigeon). For 15 species mean first arrival date in rural areas was earlier than in urban areas (significant for 11 species), while of the three species for which the reverse was true only one was significantly later. Over all species, mean first arrival date in common years was significantly earlier in rural areas by an average of three days (paired t_17_ = −3.07, p = 0.007).

**Table 1:** Basic phenological data and trends on analysed bird species.

		FAD-Rural		FAD -Urban		Paired t-test		Trend in FAD - rural				Trend in FAD - urban				Equality of trends	
Species	n_p_	Mean	SD	Mean	SD	t	p	n_r_	b	F	p	n_u_	b	F	p	F	p
Pied Wagtail	28	Feb 28	13.5	Mar 11	10.3	**−3.35**	**0.002**	28	0.09	0.07	0.786	28	- 0.70	**11.84**	**0.002**	**4.32**	**0.043**
*Motacilla alba* ^‡^																	
Woodpigeon	28	Mar 1	18.1	Feb 24	19.9	**2.33**	**0.027**	28	−1.55	**25.73**	**<0.001**	28	−1.92	**44.18**	**<0.001**	0.77	0.385
*Columba palumbus* ^‡^																	
Song Thrush	20	Mar 12	8.8	Mar 12	13.0	−0.01	0.989	28	−0.30	2.46	0.129	20	−0.86	**5.55**	**0.030**	2.11	0.153
*Turdus philomelos* ^‡^																	
White Stork	28	Mar 16	8.0	Mar 30	9.1	−**7.84**	**<0.001**	28	−0.32	3.27	0.082	28	−0.34	2.83	0.105	0.01	0.941
*Ciconia ciconia*																	
Black Redstart	25	Mar 20	7.4	Mar 25	7.9	−**2.31**	**0.030**	28	0.04	0.06	0.814	25	−0.64	**17.99**	**<0.001**	**8.48**	**0.005**
*Phoenicurus ochruros* ^‡^																	
Chiffchaff	28	Mar 25	8.1	Mar 29	7.9	−**2.61**	**0.015**	28	−0.33	3.25	0.083	28	−0.66	**23.17**	**<0.001**	2.11	0.153
*Phylloscopus collybita* ^‡^																	
Serin	26	Apr 1	10.1	Apr 2	14.9	−0.34	0.740	27	0.07	0.08	0.784	27	−0.63	3.70	0.066	2.90	0.095
*Serinus serinus* ^‡^																	
Swallow	25	Apr 1	6.1	Apr 8	6.3	−**5.88**	**<0.001**	28	−0.36	**7.56**	**0.011**	25	−0.60	**40.56**	**<0.001**	2.31	0.135
*Hirundo rustica*																	
Willow Warbler	20	Apr 5	6.5	Apr 9	6.2	−**2.22**	**0.039**	28	−0.24	3.01	0.095	20	−0.36	**5.26**	**0.034**	0.36	0.554
*Phylloscopus trochilus*																	
Blackcap	25	Apr 8	8.3	Apr 6	14.0	0.68	0.502	27	−0.48	**6.45**	**0.018**	26	−0.96	**9.59**	**0.005**	1.87	0.178
*Sylvia atricapilla* ^‡^																	
Lesser Whitethroat	24	Apr 15	4.4	Apr 17	4.9	−**2.49**	**0.020**	27	−0.06	0.03	0.592	25	−0.20	3.15	0.089	0.81	0.372
*Sylvia curruca*																	
House Martin	28	Apr 16	7.3	Apr 21	5.4	−**3.89**	**0.001**	28	−0.40	**6.54**	**0.017**	28	−0.39	**14.47**	**<0.001**	0.00	0.986
*Delichon urbica*																	
Redstart	22	Apr 19	6.3	Apr 19	11.4	−0.15	0.882	28	−0.47	**11.04**	**0.003**	22	−0.78	**7.37**	**0.013**	1.07	0.306
*Phoenicurus phoenicurus*																	
Common Whitethroat	20	Apr 23	4.8	Apr 28	6.2	−**5.36**	**<0.001**	26	−0.14	1.63	0.214	21	−0.58	**28.76**	**<0.001**	**7.61**	**0.009**
*Sylvia communis*																	
Nightingale	28	Apr 23	5.4	Apr 23	5.6	−0.93	0.359	28	−0.46	**26.93**	**<0.001**	28	−0.33	**7.79**	**0.010**	0.88	0.352
*Luscinia megarhynchos*																	
Cuckoo	28	Apr 25	4.7	Apr 27	5.6	−**2.70**	**0.012**	28	−0.22	**4.52**	**0.043**	28	−0.26	**4.62**	**0.041**	0.07	0.791
*Cuculus canorus*																	
Icterine Warbler	22	May 6	4.1	May 10	7.6	−**2.45**	**0.023**	25	−0.22	**4.96**	**0.036**	24	−0.75	**38.46**	**<0.001**	**11.04**	**0.002**
*Hippolais icterina*																	
Spotted Flycatcher	14	May 7	5.4	May 7	5.3	0.15	0.885	24	−0.21	2.99	0.098	16	−0.10	0.26	0.618	0.21	0.649
*Muscicapa striata*																	

Mean first arrival dates differed significantly between rural and urban environments for those species where paired t-test results are shown in bold. Trends in FAD were significant for those species/environments whose F-test results are shown in bold. Trends differed significantly between rural and urban environments for those species whose equality of trend F-test results are shown in bold.

Explanations: numbers of common years in the paired comparison (n_p_), mean first arrival dates (FAD) and standard deviation (SD, days) in rural and urban environments and the comparison of mean FADs using paired t-tests based on n_p_–1 degrees of freedom. Numbers of years of data in the regression of FAD on year for rural (n_r_) and urban (n_u_) environments, trends (b, days per annum change) and F-tests based on 1,n–2 degrees of freedom. The equality of trends in rural and urban environments is tested with an F-test based on 1, n_r_+n_u_–4 degrees of freedom.

Mean first arrival dates of the seven short distance migrants were significantly earlier than the 11 long distance migrants by an average of 29 (±7.2 SE) days in rural areas (2 sample t-test t_16_ = −4.07, p = 0.001) and 31 (±6.4) days in urban areas (2 sample t-test t_16_ = −4.88, p<0.001).

### Trends in First Arrival Dates

Of the rural observations, trends varied from −1.55 days/year to 0.09 days/year; 15 of 18 species had negative trends through time (i.e. towards earlier arrival) of which eight were significant and four approached significance (0.05<p<0.10). No positive trends were significant. Overall the mean of the rural trends was −0.309 (±0.084) days/year and was statistically significant from zero (1 sample t-test t_17_ = −3.67, p = 0.002). Urban trends ranged from −1.92 days/year to −0.10 days/year; all 18 had negative trends (14 significant, two approaching significance). The overall mean urban trend of −0.616 (±0.095) days/year was statistically significant from zero (1 sample t-test t_17_ = −6.47, p<0.001).

Trends were more negative in urban areas for 15 of the 18 species. Formal tests of equality of slopes showed that slopes were significantly different for four species (all more negative in urban areas) namely icterine warbler, pied wagtail, black redstart and common whitethroat. Overall the mean slope from urban areas was significantly different (more negative) than rural areas (paired t_17_ = 4.58, p<0.001), indicating greater trends to earlier arrival in urban environments.

**Figure 2:**
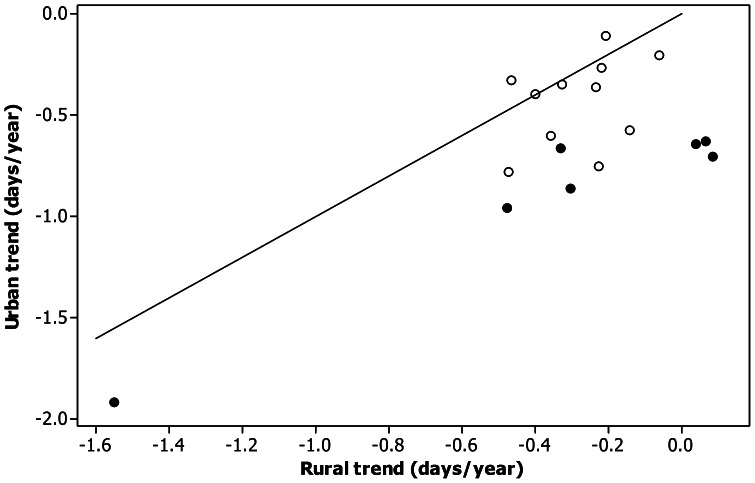
The relationship between rural and urban trends in first arrival dates in Western Poland 1983–2010. Points below the line represent species whose migration trend was stronger (more negative) in urban areas than rural areas and *vice versa*. Solid symbols represent short distance migrants, open symbols long distance migrants.

**Figure 3:**
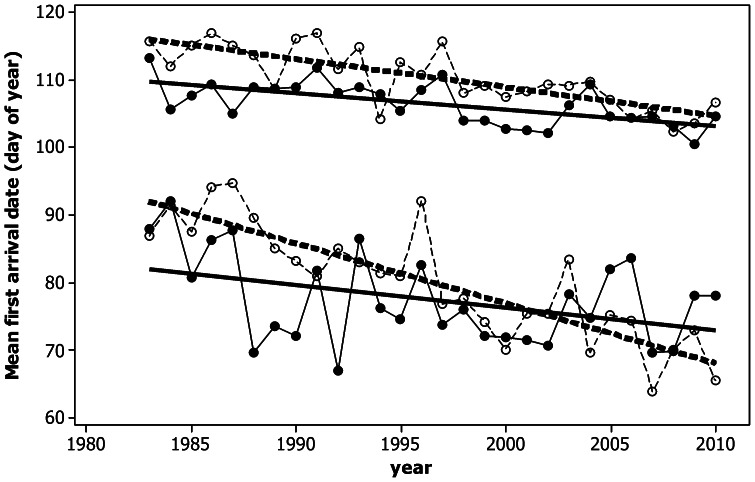
First arrival dates for rural (solid symbols and lines) and urban observations (open symbols and dotted lines) averaged over seven short distance migrants (lower part of graph) and 11 long distance migrants (upper part of graph) in Western Poland 1983–2010. Regression lines superimposed. Note than the same set of species was included in all years and in both habitats.

For rural observations there was no significant difference between trends for short and long distance migrants (2 sample t-test t_16_ = −0.39, p = 0.70) but for urban observations, trends were significantly more negative for short distance migrants (2 sample t-test t_16_ = −3.00, p = 0.008, Figure 2). These conclusions remained unchanged if wood pigeon (the greatest advance in FAD shown in Figure 2) was excluded (p = 0.172 and p = 0.007), or when nonparametric Kruskal Wallis tests (p = 0.751 and p = 0.006) were used. Figure 3 shows mean time series for short and long distance migrants for both urban and rural habitats. All trends were statistically significant, i.e. towards earlier arrival (short distance rural migrant p = 0.034, all others p<0.001). The difference in these slopes for long distance migrants between urban and rural areas was almost significant (F_1,52_ = 3.96, p = 0.052) while the difference in the trends for short distance migrants was much more distinct (F_1,52_ = 9.72, p = 0.003).

## Discussion

Recently many studies have indicated that rural and urban populations of birds differ from one another [2], [4]–[6], [11]. The main finding of our study, i.e. differences in arrival timing, also supports this view. Surprisingly, urban populations generally arrived later, although these effects differed among species.

However, the most interesting finding is that urban birds have recently advanced their arrival dates more than rural ones. This raises speculation as to the reasons, and suggests that a more in-depth study may be justified. Our study indicates that urbanization may significantly affect the phenology of these particular bird species, and consequently probably also the general biodiversity in cities. Furthermore, because of rapid urbanization worldwide and certain similarities in environmental effects between urbanisation and climate change [3], [9], we believe that our findings may have a much wider relevance.

Similarly, we find only limited evidence that migration distance influences the pattern of urban and rural advances. Generally, populations of long-distance migrants are declining more rapidly than short-distance and resident species, and may be more vulnerable or more exposed to environmental change [4]–[6], [21], [23]. Moreover, in cities some species that are migratory in rural habitats now show a strong tendency to be sedentary in urban habitats [2], [23], [26].The difference in magnitude of trends between urban and rural habitats could be related to either phenotypic plasticity or evolutionary adaptation. However, differentiating between these two options would be impossible without a controlled experimental study, although even observational data may support competing points of view [24]. An alternative suggestion is that migrants in rural surroundings have advanced their arrivals as much as phenotypic plasticity will allow, and those of urban habitats are now catching up. The magnitude of the urban heat island effect in these small cities is likely to be small [27]. Changes in FADs may thus also reflect differences in habitat selection or environmental improvement/degradation rather than changes in migration or the effects of rising temperature.

Obviously our data were collected only in one region, with relatively small cities by world standards. One major requirement of studies to identify changes in phenology is access to good long-term data. Because more detailed data are much rarer, FAD are a commonly used measure in avian phenological studies (for discussion on pros and cons see: [7], [25], [26]) and have been traditionally used as an indication of the migration timing of birds. Generally our results match an increasing body of evidence from across Europe that migrant birds are returning earlier and we have discussed this in a previous paper [26]. However, data collected specifically from urban areas are relatively rare and we believe this is the first study to explicitly compare arrivals in urban and rural areas in close proximity. Data were collected in the same way, and without directional bias to either habitat.

In conclusion, our results suggest a difference in timing of spring arrival to breeding grounds, and differences between trends in timing. Thus, we should expect a convergence between urban and rural populations or even the former overtaking the latter. Changes to the phenology of migrant birds in urban heat islands may be an analogue of climate-induced changes in the wider environment.

## Materials and Methods

### Study Area and Data Sources

Observations on the first arrival dates (FAD) of 18 migrant bird species were carried out in the southern part of the Wielkopolska and Ziemia Lubuska regions (Western Poland). Annual observations from 1983 to 2010 were recorded mainly by members of local birdwatching clubs. For more details and discussion on the accuracy of methods see [26]. Data were divided into those originating from urban (three cities, with a typical dense structure of buildings, factories and roads, covered mainly by hard (sealed) surfaces, and with populations exceeding 75,000 inhabitants: Zielona Góra, Leszno, Ostrów Wielkopolski) and rural environments (the rest of the area, covered mainly by farmland). Records were restricted to those of birds using the area for example for foraging, resting or singing; not of birds flying high over the area.

In the study area, rural habitats cover a larger area than urban habitats, but more of the observers live in the latter. Consequently, we do not believe there to be a marked difference in sampling effort between the two. FAD was used as the measure of migration phenology, partly because most alternatives can only be derived if the whole migration period is constantly monitored, but mainly because it has been shown to characterise the migratory patterns of birds especially if using data from broad citizen science studies [26], [28]. We reduced potential bias in the data by restricting observations to those of the twenty most active birdwatchers and there was no significant bias in their urban:rural ratios of observations (based on chi-squared tests per observer on the number of observations in each type of environment; χ^2^ with Yates correction = 0.409, p = 0.522). Since this was a purely observational study, no permission was required for fieldwork.

Birds were categorised according to their migratory distance; seven short-distance species wintering mainly in Western Europe and the Mediterranean basin and 11 long-distance species wintering south of the Sahara, i.e. tropical migrants [26]. Similar to other citizen science studies we have made no attempt to control for phylogenetical effects on FAD [28], [29]. However, the bulk of our analyses are paired comparisons therefore its effect will be the same in both habitats.

#### Statistical analysis

FAD for each species, year and environment was determined as the earliest of all the relevant observations. Dates were converted to days of the year (DOY) prior to analysis, such that that April 1 =  DOY 91 or DOY 92 in a leap year. Mean FADs were compared between rural and urban environments for each species using paired t-tests based on years common to both environments. Trends in FADs for each species and environment were estimated from regressions of FAD on year. The resulting coefficients are estimates of the changes (in days/year) that have occurred, and are of greater interest to us, in this instance, than their statistical significance. Differences between trends in urban and rural areas for individual species were tested using standard equality of slope tests based on ANCOVA [30].

Mean time series for short and long distance migrants for each environment were calculated as averages of all available data and analysed as above. Paired, one sample and two sample t-tests (as appropriate) were used as described in the results to compare overall differences between urban and rural areas, and to compare summary variables with migratory distance. Data were tested for normality using Kolmogorov-Smirnov tests and where non-normality was detected appropriate nonparametric tests were used to confirm conclusions.

All statistical analyses were conducted using MINITAB v.16.
